# Recurrent Hyperhemolysis Syndrome in Sickle Cell Disease

**DOI:** 10.7759/cureus.14991

**Published:** 2021-05-12

**Authors:** Rafey Rehman, Saad B Saadat, Deanna H Tran, Sinziana Constantinescu, Yusuf Qamruzzaman

**Affiliations:** 1 Hematology and Oncology, Oakland University William Beaumont School of Medicine, Rochester Hills, USA; 2 Anesthesiology, Beaumont Health, Royal Oak, USA; 3 Hematology and Oncology, Beaumont Health, Royal Oak, USA

**Keywords:** hyperhemolysis syndrome, sickle cell, transfusion reaction, hemoglobin, anemia

## Abstract

Sickle cell disease is a disorder of hemoglobin. The abnormal hemoglobin S disrupts blood flow, thereby resulting in acute painful sickle cell crisis. These episodes frequently prompt packed red blood cell transfusions to replace a patient’s functional hemoglobin stores. Production of alloantibodies and autoantibodies to these transfusions can result in a rare, but serious, complication known as hyperhemolysis syndrome. Hyperhemolysis syndrome presents several challenges in regard to its acute management and the consequent difficulties in finding future compatible blood products. We report a case of recurrent hyperhemolysis syndrome. Both episodes occurred following orthopedic procedures, and the recurrent episode proved refractory to multiple treatments.

## Introduction

Sickle cell disease (SCD) is an inherited hemoglobin disorder, resulting in homozygous hemoglobin S [1]. Patients often require lifelong transfusions of packed red blood cells (pRBCs). In addition to treating acute events such as sickle cell crisis, transfusions can also reduce perioperative complications in SCD patients [2]. These recurrent transfusions compound additional risks, particularly hyperhemolysis syndrome (HS), which can exacerbate ongoing hemolysis and a patient’s anemia [1].

The pathophysiology of HS remains unclear. Proposed mechanisms include bystander hemolysis [3], macrophage activation [4], suppression of erythropoiesis [5], and oxidative stress or complement-mediated destruction of red blood cells (RBCs) [6]. Prior studies show that further transfusions paradoxically exacerbate the resulting anemia through the destruction of both host and donor RBCs [3,7]. Recurrent episodes of HS are particularly rare, with past reported cases in two adults [5] and two children [4,8]. Risk factors for recurrent HS and possible differences between primary and recurrent episodes of HS also remain largely unknown. Some studies in the literature report that patients with a new alloantibody are more likely to tolerate further transfusions during active episodes of HS [9].

The current standard treatment for HS includes intravenous immune globulin (IVIG), steroids, and preferential avoidance of further transfusions of RBCs [2,4,10]. For refractory cases, antimacrophage therapy such as tocilizumab may be beneficial [11]. For recurrent cases, plasma-to-RBC exchange transfusion with concurrent standard care may be successful [12].

## Case presentation

A 47-year-old female patient with a history of hemoglobin-SC anemia was admitted for an infected chronic right hip wound. She was evaluated by the orthopedic surgery service and recommended removal of her hip prosthesis with antibiotic spacer placement. On initial presentation, her hemoglobin was 9.4 g/dL, mean corpuscular volume (MCV) was 73 fL, and platelet count was 140 bil/L. Her kidney function and liver function were normal. A hemoglobin electrophoresis prior to surgery showed a hemoglobin S of 48.6% and hemoglobin C of 46.2%. She received three units of pRBCs over the course of five days prior to surgery. Her hemoglobin post-operatively declined to 6.9 g/dL. She received an additional two units of pRBCs, and her hemoglobin stabilized to her baseline of 8-9 g/dL. On post-operative day 9 (nine days after her initial transfusion), her hemoglobin suddenly dropped to 2.5 g/dL and her lactase dehydrogenase (LDH) rose above 4,500 U/L, due to suspected HS. She became hemodynamically unstable, prompting intubation. She was started on IVIG, methylprednisolone, and tocilizumab. Although additional transfusions of pRBCs were avoided in the initial medical management, further transfusions were indicated for her hemodynamic instability and lactic acidosis (lactic acid of 22.2 mmol/L). Her hemoglobin would respond to the transfusions with transient elevations, but then decline the following day. Due to her labile hemoglobin and deteriorating clinical condition, further therapies were sought. She was started on darbepoetin. She was also noted to have splenomegaly, and this was felt to play a role in her HS. She subsequently underwent splenic embolization with Interventional Radiology. Despite all treatments, her hemodynamic instability persisted alongside clinical evidence of hypoxia, thereby necessitating daily transfusions of pRBCs. She developed renal failure requiring dialysis, and acute liver failure (aspartate aminotransferase [AST] peaked at 5,982 U/L and total bilirubin was 14.6 mg/dL). As a final resort, she underwent plasmapheresis followed by rituximab. Despite all efforts, her condition continued to deteriorate, and eventually 12 days after her initial onset of HS she passed away.

A review of her records revealed that she had a prior history of HS, which had also followed hip surgery 12 years earlier. In this previous episode, her hemoglobin dropped to 3 g/dL. Transfusions were withheld, and she was treated with steroids and IVIG. After about two weeks, her hemoglobin returned back to her baseline.

## Discussion

We presented a case of a 47-year-old female with hemoglobin-SC anemia who was diagnosed with HS nine days after transfusion of pRBCs. She had a previous episode of HS 12 years prior, also after hip surgery. On that occasion, transfusions were withheld. She was treated with steroids and IVIG, and her hemoglobin returned to baseline after about two weeks. On the more recent occurrence, she developed severe anemia after resection of right total hip arthroplasty and placement of prosthesis with antibiotic-loaded acrylic cement, nine days after initial transfusion. Prior to her surgery, she received three units of pPRBCs. Given her history of SCD, positive titers for anti-IgM and anti-C3D, marked increase in splenomegaly, and previous episode of HS, HS secondary to SCD was felt to be the most likely diagnosis. Initial treatments included IVIG 0.4 mg/kg daily for five days, methylprednisolone 125 mg every 12 hours, three doses of tocilizumab, and darbepoetin, which yielded no clinical improvement. Due to hemodynamic instability, she required further transfusions. She would transiently respond to these transfusions with increased hemoglobin up to 5.5, but this would eventually trend downwards to <3.0. Due to a lack of response, splenic artery embolization was also performed to decrease RBC sequestration. Plasmapheresis and rituximab were also given as a last resort. Despite all efforts, the patient unfortunately passed away.

HS is an uncommon but severe complication following transfusions of pRBCs, which is found especially in patients with SCD. Risk is increased in patients with a history of multiple transfusions [13]. The rate of incidence of HS in SCD patients is estimated to range from 1% to 19% [8]. HS typically presents about seven days after transfusion with pain, fever, jaundice, and hemoglobinuria [1]. Lab results would show severe hemolytic anemia with increased LDH, hyperbilirubinemia, and possible reticulocytopenia [8]. Antibodies against RBCs are typically not detected in patient samples, and patients may still be at risk of HS despite screening for ABO, Rh-factor, and K-incompatibilities. HS is believed to be caused by the destruction of donor and host RBCs [4]. The exact mechanism of HS is unclear, as there are multiple proposed mechanisms for its pathogenesis. One proposed mechanism of HS is known as bystander hemolysis. Petz et al. describe this phenomenon as immune-mediated hemolysis of cells that do not express antigens that are positive in the patient’s serum [5]. It is thought that antibodies play a role in the pathogenesis of HS despite a lack of reactive antigens to these antibodies. Another proposed mechanism for the pathogenesis of HS is the suppression of erythropoiesis. This is characterized by reticulocytopenia with a decreased post-transfusion hemoglobin and a clinical picture of HS. It is hypothesized repetitive transfusions of pRBCs may cause reactive inhibition of erythropoiesis that possibly exacerbates post-transfusion anemia [4]. Additionally, it has been hypothesized that an increased activation of macrophages may contribute to the anemia seen in HS. When sickled, RBCs express high levels of antigens, such as phosphatidylserine, and high levels of IgG on their cell surface to potentiate recognition from hyperactivated macrophages, leading to extravascular hemolysis [13].

The mainstay treatment for HS is steroids, IVIG, and restricting blood transfusions to hemodynamically stable patients. Mild cases of HS can receive steroids with close monitoring of hemoglobin [13]. IVIG, erythropoietin, and steroids have been used in the treatment of HS, in light of evidence suggesting that steroids and IVIG have a synergistic effect in suppressing macrophages [4].

## Conclusions

In conclusion, HS is a rare, severe, post-transfusion complication that should be considered in the event of severely decreased hemoglobin following transfusions of pRBCs in patients with SCD. There are few documented cases of recurrent HS. Our case supports the hypothesis that a history of HS may increase the risk of HS with future transfusions. The recommended treatment plan is avoidance of further transfusions, and administration of IVIG and methylprednisolone. Other strategies involving tocilizumab, rituximab, and splenic embolization have been attempted with unclear benefit.
